# Autoimmune Hepatitis-Primary Biliary Cholangitis Overlap Syndrome With Limited Systemic Sclerosis

**DOI:** 10.7759/cureus.113525

**Published:** 2026-07-28

**Authors:** Cesar Fabian Vallejo Rico, Guadalupe de los Angeles Salazar Gonzalez, Jaqueline Reyes Rueda, Valeria Gonzalez Quiroz, Jorge Alberto Garay Hernandez

**Affiliations:** 1 Internal Medicine, Mexican Social Security Institute, Torreón, MEX; 2 Internal Medicine, Mexican Social Security Institute, Durango, MEX; 3 Rheumatology, Mexican Social Security Institute, Torreón, MEX; 4 Internal Medicine, Instituto Mexicano del Seguro Social, Durango, MEX

**Keywords:** autoimmune hepatitis (aih), limited scleroderma, percutaneous liver biopsy, primary biliary cholangitis (pbc), raynaud’s disease

## Abstract

Autoimmune hepatitis-primary biliary cholangitis (AIH-PBC) overlap syndrome is an uncommon autoimmune liver disease, and its association with limited systemic sclerosis is rarely reported. We describe the case of a 41-year-old woman with a history of intrahepatic cholestasis of pregnancy (ICP) who presented with chronic pruritus, arthralgias, recurrent urinary tract infections, and Raynaud phenomenon. Laboratory evaluation demonstrated a predominantly cholestatic liver profile, positive antimitochondrial antibodies (AMAs), high-titer antinuclear antibodies (ANA) with a centromere pattern, elevated immunoglobulin G (IgG), and negative viral serologies. Imaging revealed cirrhotic liver morphology, splenomegaly, and features of clinically significant portal hypertension. Liver biopsy showed interface hepatitis with lymphoplasmacytic infiltrates, florid duct lesions, and ductopenia, supporting the diagnosis of AIH-PBC overlap syndrome in conjunction with the clinical and serological findings. Positive anticentromere antibodies confirmed limited systemic sclerosis. Despite treatment with ursodeoxycholic acid and immunosuppressive therapy, the patient experienced progressive liver dysfunction, developing decompensated cirrhosis with ascites and worsening portal hypertension, and is currently undergoing liver transplant evaluation.

This case highlights the importance of recognizing extrahepatic autoimmune manifestations and integrating clinical, serological, and histopathological findings to establish an early diagnosis of AIH-PBC overlap syndrome associated with limited systemic sclerosis, allowing timely treatment and referral for liver transplantation in patients with progressive disease.

## Introduction

Autoimmune liver diseases comprise a heterogeneous group of immune-mediated disorders that include autoimmune hepatitis (AIH), primary biliary cholangitis (PBC), and primary sclerosing cholangitis (PSC). Although traditionally considered distinct entities, they may coexist in the same patient, giving rise to overlap syndromes characterized by combined clinical, biochemical, serological, and histopathological features [1,2].

Among these conditions, AIH-PBC overlap syndrome is the most frequently recognized overlap phenotype but remains uncommon. Increasing evidence suggests that overlap syndromes represent a phenotypic continuum of autoimmune liver disease rather than separate disease entities, reflecting shared mechanisms of immune dysregulation and genetic susceptibility [2,3].

Diagnosis requires integration of clinical presentation, liver biochemistry, autoantibody profile, imaging studies, and histopathological findings because no single test is diagnostic. Current European Association for the Study of the Liver (EASL) guidelines recommend liver biopsy when AIH is suspected in patients with cholestatic liver disease, and the Paris criteria remain the preferred diagnostic framework for AIH-PBC overlap syndrome [4,5].

PBC has a well-recognized association with limited systemic sclerosis, particularly in patients with anticentromere antibody positivity, suggesting common immunopathogenic mechanisms between both disorders. Consequently, persistent cholestatic liver abnormalities in these patients should prompt evaluation for coexisting autoimmune liver disease [6,7].

We report a rare case of AIH-PBC overlap syndrome associated with limited systemic sclerosis, in which recurrent intrahepatic cholestasis of pregnancy (ICP) preceded the diagnosis of chronic autoimmune liver disease. Despite combination therapy, the patient progressed to cirrhosis with portal hypertension requiring liver transplant evaluation. This case highlights the diagnostic challenges of this uncommon association and underscores the importance of integrating clinical, serological, and histopathological findings to achieve an early diagnosis and optimize management.

## Case presentation

A 41-year-old woman with a family history of rheumatoid arthritis in her brother presented for evaluation. Her obstetric history was notable for ICP during pregnancies in 2015 and 2017, characterized by jaundice, generalized pruritus, and acholic stools, with complete resolution after delivery and no subsequent follow-up. She denied tobacco use, alcohol consumption, illicit drug use, oral contraceptive pill use, chronic medication use, and herbal supplement intake.

**Figure 1:**
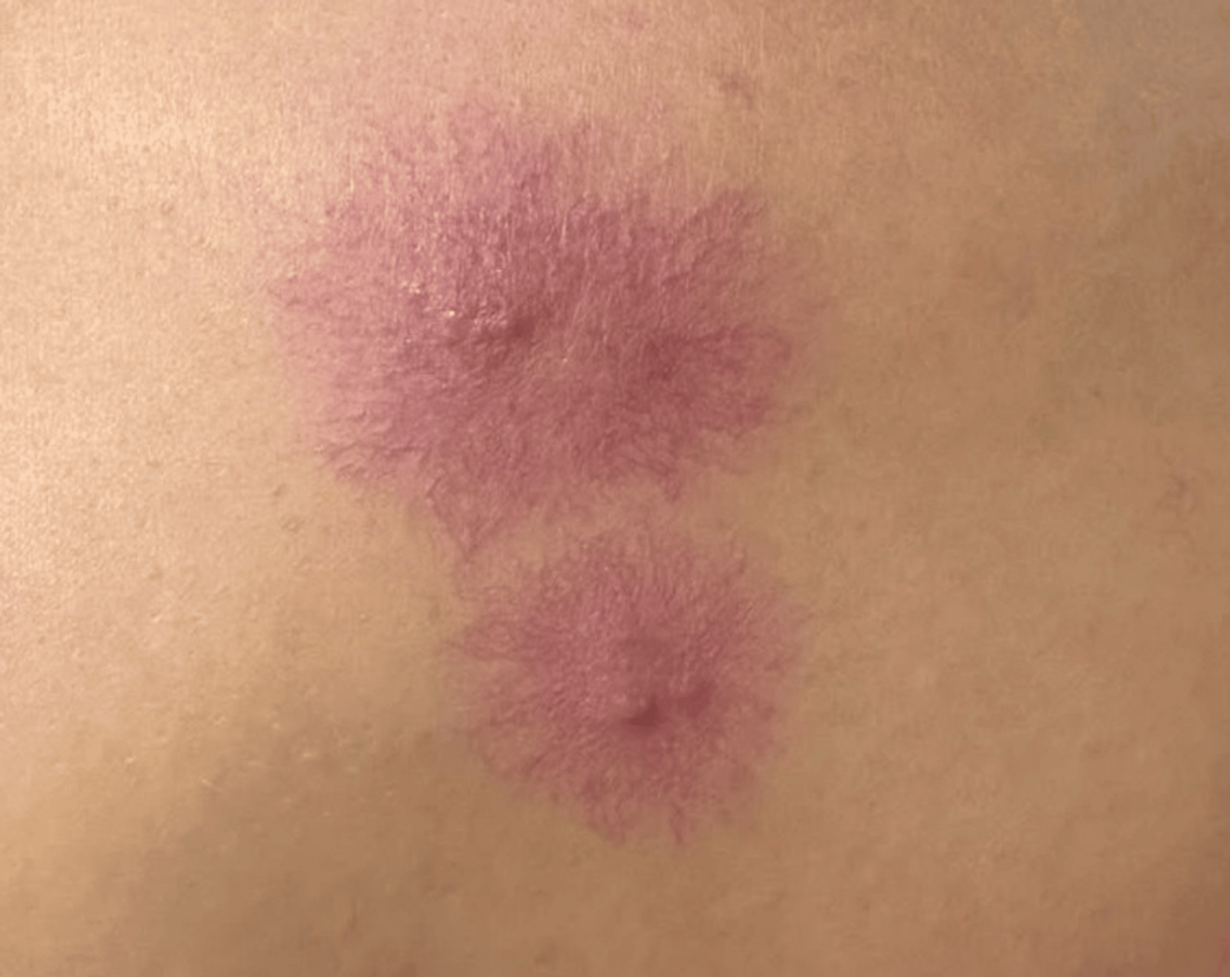
Cutaneous telangiectasias on the upper chest in a patient with AIH-PBC overlap syndrome and limited systemic sclerosis Clinical photograph demonstrating multiple arborizing telangiectasias over the anterior upper chest. These cutaneous vascular lesions are consistent with the patient's diagnosis of limited systemic sclerosis (anticentromere antibody-positive) and were documented during the initial clinical evaluation. AIH: Autoimmune hepatitis; PBC: Primary biliary cholangitis

In 2022, she developed dysuria, urinary urgency, and tenesmus in the setting of recurrent urinary tract infections. A directed review of systems revealed chronic generalized pruritus for approximately one year, as well as arthralgia involving the small joints of the hands and wrists. Physical examination was remarkable only for monophasic Raynaud phenomenon, telangiectasias on face, anterior and posterior thorax, accompanied by pruritus with bleeding upon scratching, which pale upon pressure and skin induration involving both feet (Figure 1).

Initial laboratory evaluation revealed thrombocytopenia and abnormal liver biochemical tests characterized by elevated aminotransferases, a cholestatic enzyme pattern, and mild hyperbilirubinemia (Table 1). Given these findings, abdominal ultrasonography was performed to evaluate for portal hypertension and underlying hepatobiliary disease. Imaging demonstrated increased hepatic echogenicity, a nodular liver surface, splenomegaly, and normal-caliber bile ducts without evidence of cholelithiasis, findings suggestive of chronic liver disease with portal hypertension.

**Table 1:** Initial laboratory findings ALP: Alkaline phosphatase; LDH: Lactate dehydrogenase; dsDNA: Double-stranded DNA; ANA: Antinuclear antibodies; IgG: Immunoglobulin G; HIV: Human immunodeficiency virus; AMA: Antimitochondrial antibody; LKM: Liver kidney microsomal antibody

Parameter	Result	Reference range	Units
Hemoglobin	12.5	12.5-16.8	g/dL
Hematocrit	38.90	36-47	%
Platelet count	88	142-424	10^3^/µL
Total cholesterol	196	10-200	mg/dl
Albumin	4.1	3.5-5	g/dL
Aspartate aminotransferase	109	8-37	UI/L
Alanine aminotransferase	134	1-41	UI/L
ALP	514	38-126	UI/L
LDH	245	120-246	uL
Gamma glutamyl transferase	225	12-58	UI/L
Total bilirubin	1.5	0-1.1	mg/dl
Direct bilirubin	1	0-0.20	mg/dl
Indirect bilirubin	0.5	0-0.90	mg/dl
Anti-dsDNA	< 1:10	Minor 27	IU/ml
ANA	Positive 1: 1280	Positive/Negative	Title
Nuclear membrane pattern	0.15	Positive/Negative	Title
Centromere pattern	1:160, fine granular nuclear pattern	Positive/Negative	Pattern
Complement C4	12.4	10-40	mg/dl
IgG	1549	700-1500	mg/dl
Hepatitis B virus serology	Negative	0.90-1.0	S/CO
HIV serology	Negative	0.90-1.0	S/CO
Hepatitis C virus serology	Negative	0.90-1.0	S/CO
AMA	Positive	Positive/Negative	Title
Anti-LKM antibody	Negative	Positive/Negative	Title
Anti-smooth muscle	Negative	Positive/Negative	Title

Based on these findings, the Fibrosis-4 Index (FIB-4 Index) score was calculated at 5.1 (F3-F4), consistent with advanced fibrosis, and the patient was diagnosed with compensated chronic liver disease (Child-Pugh class A). An etiological workup was subsequently performed (Table 1).

Serologic testing showed antinuclear antibodies (ANA) positive at a titer of 1:1,280 with a centromere pattern, antimitochondrial antibody (AMA) positivity, immunoglobulin G (IgG) levels at the upper limit of normal, and negative viral hepatitis and human immunodeficiency virus (HIV) serologies.

**Figure 2:**
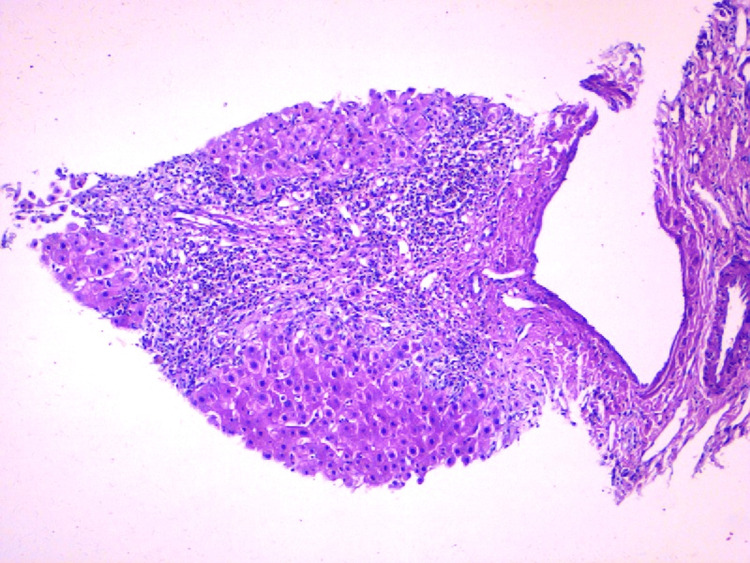
Liver biopsy, H&E stain, high-power field (×100) Histologic section demonstrating expansion of the portal tract by a dense predominantly mononuclear inflammatory infiltrate with scattered plasma cells. The inflammatory infiltrate extends beyond the limiting plate into the periportal hepatic parenchyma, consistent with interface hepatitis. H&E: Hematoxylin and eosin

**Figure 3:**
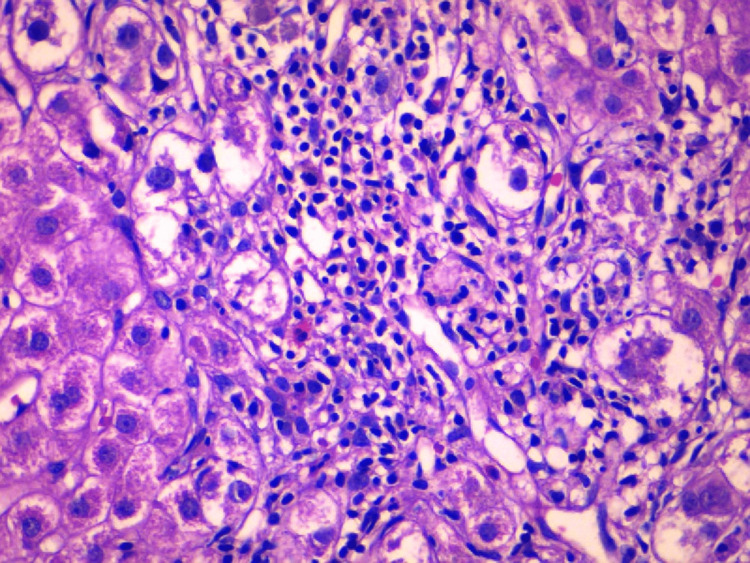
Liver biopsy, H&E stain, high-power field (×400) Representative high-power field showing interface hepatitis with a predominantly lymphoplasmacytic inflammatory infiltrate and associated hepatocellular injury. Correlation with the remaining biopsy sections demonstrated concomitant bile duct injury, supporting the histopathologic diagnosis of AIH-PBC overlap syndrome. H&E: Hematoxylin and eosin; AIH: Autoimmune hepatitis; PBC: Primary biliary cholangitis

Collectively, these findings raised a strong suspicion of AIH according to the Simplified International Autoimmune Hepatitis Group criteria. However, the predominantly cholestatic biochemical profile, persistent pruritus, previous history of ICP, and positive AMA suggested an AIH-PBC overlap syndrome according to the Paris criteria. Following completion of the autoimmune serologic workup in December 2022, treatment with ursodeoxycholic acid, deflazacort, and azathioprine was initiated because of the high clinical suspicion of AIH-PBC overlap syndrome. The patient experienced progressive resolution of pruritus and improvement in liver biochemical abnormalities. Because of thrombocytopenia and the associated bleeding risk, liver biopsy was deferred. Once the procedure was considered safe, an ultrasound-guided percutaneous liver biopsy was performed in March 2023 to obtain histopathological confirmation of the diagnosis. Histopathological examination demonstrated interface hepatitis with dense lymphoplasmacytic infiltrates, florid duct lesions, and ductopenia, findings consistent with AIH-PBC overlap syndrome (Figures 2, 3).

Given the presence of Raynaud phenomenon, telangiectasias, skin induration and the centromere ANA pattern, limited systemic sclerosis was also suspected. Positive anticentromere antibodies subsequently confirmed the diagnosis, and mycophenolate mofetil was added to the existing treatment regimen.

Persistent thrombocytopenia and splenomegaly raised concern for clinically significant portal hypertension. Upper gastrointestinal endoscopy revealed small esophageal varices.

Despite treatment with ursodeoxycholic acid and immunosuppressive therapy, the patient experienced progressive liver disease with worsening biochemical parameters (Table 2), development of peripheral edema, ascites, and clinically significant portal hypertension. She progressed to decompensated cirrhosis (Child-Pugh class C) with a Model for End-Stage Liver Disease (MELD) score of 18 and is currently undergoing evaluation for liver transplantation.

**Table 2:** Evolution of laboratory parameters during follow-up ALP: Alkaline phosphatase; IgG: Immunoglobulin G

Parameter	2022	2023	2025	2026	Reference range	Units
Aspartate aminotransferase	111	115	170	133	8-37	UI/L
Alanine aminotransferase	127	96	131	111	1-41	UI/L
ALP	273	306	679	230	38-126	UI/L
Gamma glutamyl transferase	332	260	423	113	12-58	UI/L
Total bilirubin	2.4	1.9	4.3	4.9	0-1.1	mg/dl
Albumin	4.8	3.8	2.5	2.3	3.5-5.0	mg/dL
IgG	1,549	-	-	1,687	700-1,500	mg/dL
Platelet count	76	75	76	83	142-424	10^3^/µL

## Discussion

Autoimmune liver diseases constitute a spectrum of immune-mediated disorders in which clinical, serological, biochemical, and histopathological features may overlap, making diagnosis particularly challenging. In our patient, the coexistence of a cholestatic biochemical profile, AMA positivity, elevated IgG, and characteristic histological findings supported the diagnosis of AIH-PBC overlap syndrome. Rather than relying on a single diagnostic test, current evidence emphasizes that diagnosis should be based on an integrated clinicopathological assessment that incorporates clinical presentation, laboratory findings, imaging studies, autoantibody profile, and liver histology [1,4].

The diagnosis of AIH-PBC overlap syndrome remains difficult because individual clinical or histopathological findings are not disease-specific. Interface hepatitis, though considered the hallmark of AIH, may also be observed in PBC, whereas cholangitic lesions can coexist with AIH in overlap syndromes. Consequently, histological findings should always be interpreted within the appropriate clinical and immunological context. Although the Paris criteria remain the most widely accepted diagnostic framework, they should complement rather than replace comprehensive clinical judgment because overlap phenotypes represent a continuum rather than completely distinct disease entities [3,4].

Liver biopsy played a pivotal role in our patient. Although biopsy is not routinely required for the diagnosis of PBC, current guidelines recommend its performance whenever AIH is suspected or when overlap syndrome cannot be confidently established by serological and biochemical findings alone [5,8]. In this case, histopathological examination demonstrated interface hepatitis together with florid duct lesions and ductopenia, confirming the coexistence of hepatitic and cholangiopathic injury and allowing definitive classification as AIH-PBC overlap syndrome.

Cross-sectional imaging also contributed to the diagnostic evaluation by excluding biliary obstruction and other cholangiopathies before liver biopsy was performed. Although imaging lacks specificity for AIH, it remains essential in the diagnostic algorithm because it excludes alternative causes of cholestatic liver disease, particularly PSC and extrahepatic biliary obstruction, thereby increasing the diagnostic value of histopathological findings [9,10].

Another remarkable feature of this case is the coexistence of limited systemic sclerosis. PBC is the autoimmune liver disease most frequently associated with systemic sclerosis, particularly in patients with the limited cutaneous subtype and anticentromere antibody positivity. In contrast, AIH is considerably less common in this population, making AIH-PBC overlap syndrome associated with limited systemic sclerosis an unusual clinical presentation. This association is thought to result from shared mechanisms of immune dysregulation, common genetic susceptibility loci, and overlapping profibrotic pathways rather than from independent autoimmune diseases occurring by chance [6,11].

Our patient presented with Raynaud phenomenon, telangiectasias, positive ANA with a centromere pattern, and subsequent anticentromere antibody positivity, findings that supported the diagnosis of limited systemic sclerosis. Recognition of these extrahepatic manifestations was clinically relevant because autoimmune liver diseases frequently coexist with systemic rheumatic disorders, and identifying both conditions influences long-term surveillance, multidisciplinary management, and therapeutic decision-making [7,12].

An additional aspect that distinguishes this case is the patient's history of ICP several years before the diagnosis of autoimmune liver disease. Although a direct causal relationship cannot be established, this obstetric history may represent the earliest clinical manifestation of an underlying predisposition to immune-mediated cholestatic liver disease. Epidemiological evidence indicates that women with previous ICP have a substantially increased long-term risk of developing hepatobiliary disorders, supporting the hypothesis that pregnancy-associated cholestasis may unmask latent autoimmune cholestatic diseases in genetically susceptible individuals [13]. In our patient, the occurrence of ICP preceded the subsequent development of AIH-PBC overlap syndrome, raising the possibility that pregnancy represented the first clinical trigger revealing an underlying autoimmune process. Recognition of this association is clinically relevant because women with a history of ICP who later develop persistent cholestatic liver test abnormalities or autoimmune manifestations may benefit from earlier evaluation for autoimmune liver disease, potentially allowing earlier diagnosis and treatment.

Current management of AIH-PBC overlap syndrome aims to treat both hepatocellular inflammation and cholestatic injury. Available evidence supports the use of ursodeoxycholic acid combined with corticosteroids and immunosuppressive agents because combination therapy provides superior biochemical and histological responses compared with ursodeoxycholic acid alone. Nevertheless, robust evidence demonstrating improved transplant-free survival remains limited, and disease progression may still occur despite appropriate treatment [3,14].

Despite receiving ursodeoxycholic acid together with immunosuppressive therapy, our patient experienced progressive liver dysfunction characterized by worsening portal hypertension, ascites, and decompensated cirrhosis requiring evaluation for liver transplantation. This unfavorable evolution is consistent with previous studies demonstrating that patients with AIH-PBC overlap syndrome have higher rates of portal hypertension, hepatic decompensation, and liver transplantation than those with isolated AIH or PBC, emphasizing the importance of early recognition and close follow-up [14,15].

Compared with previously published reports, this case contributes several noteworthy clinical observations. First, it documents the uncommon coexistence of AIH-PBC overlap syndrome with limited systemic sclerosis confirmed by anticentromere antibodies. Second, it illustrates a possible temporal relationship between ICP and the subsequent development of autoimmune cholestatic liver disease. Finally, it demonstrates that progressive hepatic decompensation may occur despite guideline-directed combination therapy, highlighting the heterogeneous clinical behavior of overlap syndromes and the importance of early referral for liver transplantation in patients with progressive disease.

Literature review and comparison with previously reported cases

To better contextualize our findings, we compared our patient with previously reported cases of AIH-PBC overlap syndrome associated with systemic sclerosis (Table 3). Although overlap syndrome and systemic sclerosis are individually well-recognized autoimmune conditions, their coexistence remains uncommon. This comparison highlights the clinical heterogeneity, immunological profile, histopathological findings, therapeutic approaches, and outcomes reported in the literature, emphasizing the distinctive features of our patient.

**Table 3:** Comparison with previously reported cases Case 1 is a part of Efe et al. [16] and Cases 2, 3, and 4 are a part of Han et al. [17]. AIH: Autoimmune hepatitis; PBC: Primary biliary cholangitis; ANA: Antinuclear antibody; AMA: Antimitochondrial antibody; IgG: Immunoglobulin G; ICP: Intrahepatic cholestasis of pregnancy

Feature	Case 1	Case 2	Case 3	Case 4	Present case
Gender	Female	Female	Female	Female	Female
Age	51 years	68 years	54 years	41 years	41 years
Type of systemic sclerosis	Limited	Limited	Limited	Diffuse	Limited
Manifestations of sclerosis	Raynaud's phenomenon, sclerodactyly	Raynaud's phenomenon, sclerodactyly	Raynaud's phenomenon, sclerodactyly	Diffuse skin thickening, sclerodactyly, Raynaud's phenomenon	Diffuse skin thickening, Raynaud's phenomenon, telangiectasias
Limited cutaneous systemic sclerosis-related antibody	Anticentromere antibody positive	Anticentromere antibody positive	Anticentromere antibody positive	ANA, anti Ro, Perinuclear anti-neutrophil cytoplasmic antibody positives	Anticentromere antibody positive
Liver diagnosis	AIH-PBC overlap syndrome	AIH-PBC overlap syndrome	AIH-PBC overlap syndrome	AIH-PBCs overlap syndrome	AIH-PBC overlap syndrome
Histological confirmation	Yes	Yes	Yes	Yes	Yes
AMA	Positive	Positive	Positive	Positive	Positive
Elevated IgG	Yes	Yes	Yes	Yes	Yes
Treatment	Ursodeoxycholic acid + corticosteroids	Ursodeoxycholic acid + immunosuppression	Ursodeoxycholic acid + corticosteroids	Ursodeoxycholic acid + azathioprine	Ursodeoxycholic acid + azathioprine+ deflazacort
Evolution	Clinical improvement	Stable	Stable	Skin and liver improvement	Progression to decompensated cirrhosis and evaluation for transplantation
Main contribution	First reported case of AIH-PBC overlap syndrome + Limited cutaneous systemic sclerosis	Association with autoimmune thrombocytopenic purpura	Association with hepatic fibrosis	First case of AIH-PBC overlap syndrome associated with diffuse systemic sclerosis	This is the first case that, in addition to AIH-PBC overlap syndrome and limited cutaneous systemic sclerosis, presents a history of ICP followed by progression to decompensated cirrhosis and evaluation for transplantation, which suggests that ICP may have represented an early manifestation of predisposition to autoimmune cholestatic disease.

## Conclusions

AIH-PBC overlap syndrome associated with limited systemic sclerosis is an uncommon autoimmune condition that requires a high index of suspicion because its diagnosis depends on integrating clinical, biochemical, serological, imaging, and histopathological findings rather than relying on a single diagnostic test. This case illustrates the pivotal role of liver biopsy in confirming overlap syndrome and guiding appropriate therapy when AIH is suspected in patients with cholestatic liver disease. The coexistence of limited systemic sclerosis and a previous history of ICP represents a particularly noteworthy combination that raises the possibility that pregnancy-associated cholestasis may constitute an early clinical manifestation of an underlying predisposition to autoimmune cholestatic liver disease in susceptible individuals. Although causality cannot be inferred from a single case, clinicians should maintain a low threshold for evaluating autoimmune liver disease in women with persistent cholestatic liver test abnormalities after ICP, particularly when accompanied by autoimmune manifestations. Finally, this case highlights that disease progression to decompensated cirrhosis may occur despite guideline-directed combination therapy, underscoring the importance of close longitudinal follow-up, multidisciplinary management, and timely referral for liver transplantation in patients with advanced disease.
